# 

**Published:** 1925

**Authors:** 


### CONTENTS.

*Page*

**University of Bristol: Royal Opening of the New Buildings.** By J. A. Nixon, C.M.G., M.D. Cantab. ... ... ... ... ... 73

**A Note on Asthma.** By J. R. Charles, M.D., F.R.C.P. ... ... 84

**Diagnosis of the Dyspepsias.** By A. Rendle Short, M.D., B.S., B.Sc., F.R.C.S. ... ... ... ... ... ... ... ... ... ... 88

**The Present Position of Surgery of the Nervous System.** By Percy Sargent, C.M.G., D.S.O., F.R.C.S. ... ... ... .. 101

**A Case of Intrathecal Extramedullary Spinal Tumour.** By A. Wilfrid Adams, M.S., F.R.C.S. ... ... ... ... ... ... ... 116

**Some Points in the Differential Diagnosis of Focal Infection in the Nasal Sinuses causing Ocular and other Systemic Infections.** By Patrick Watson-Williams, M.D. ... ... ... ... ... 121

**Reviews of Books** .. ... ... ... ... ... ... ... ... 127

**Editorial Notes** .. ... ... ... ... ... ... ... .. 138

**Meetings of Societies** ... ... .. ... ... ... ... ... 142

**Obituary:** James Taylor ... ... ... ... ... ... ... 145

Arthur Bancks Prowse, M.D., F.R.C.S. ... .. ... 149

H. L. Evans, M.B., C.M. Ed. ... ... ... ... ... 151

**The Library of the Bristol Medico-Chirurgical Society** ... ... 152

**Local Medical Notes** ... ... ... ... ... ... ... ... 154

